# Phage resistance mutations affecting the bacterial cell surface increase susceptibility to fungi in a model cheese community

**DOI:** 10.1093/ismeco/ycae101

**Published:** 2024-08-02

**Authors:** Tara C J Spencer-Drakes, Angel Sarabia, Gary Heussler, Emily C Pierce, Manon Morin, Steven Villareal, Rachel J Dutton

**Affiliations:** Division of Biological Sciences, Department of Molecular Biology, University of California, San Diego, 9500 Gilman Drive, La Jolla, CA 92093, United States; Division of Biological Sciences, Department of Molecular Biology, University of California, San Diego, 9500 Gilman Drive, La Jolla, CA 92093, United States; La Jolla Institute for Immunology, 9420 Athena Circle, La Jolla, CA 92037, United States; Division of Biological Sciences, Department of Molecular Biology, University of California, San Diego, 9500 Gilman Drive, La Jolla, CA 92093, United States; Division of Biological Sciences, Department of Molecular Biology, University of California, San Diego, 9500 Gilman Drive, La Jolla, CA 92093, United States; Arcadia Science, 3100 San Pablo Avenue, Suite #120, Berkeley, CA 94702, United States; Division of Biological Sciences, Department of Molecular Biology, University of California, San Diego, 9500 Gilman Drive, La Jolla, CA 92093, United States; Arcadia Science, 3100 San Pablo Avenue, Suite #120, Berkeley, CA 94702, United States; Division of Biological Sciences, Department of Molecular Biology, University of California, San Diego, 9500 Gilman Drive, La Jolla, CA 92093, United States; Division of Biological Sciences, Department of Molecular Biology, University of California, San Diego, 9500 Gilman Drive, La Jolla, CA 92093, United States; Astera Institute, 2625 Alcatraz Ave, #201, Berkeley, CA 94705, United States

**Keywords:** microbiome, host–phage interactions, LPS, O-antigen, cheese

## Abstract

Diverse populations of bacteriophages infect and coevolve with their bacterial hosts. Although host recognition and infection occur within microbiomes, the molecular mechanisms underlying host–phage interactions within a community context remain poorly studied. The biofilms (rinds) of aged cheeses contain taxonomically diverse microbial communities that follow reproducible growth patterns and can be manipulated under laboratory conditions. In this study, we use cheese as a model for studying phage–microbe interactions by identifying and characterizing a tractable host–phage pair co-occurring within a model Brie-like community. We isolated a novel bacteriophage, TS33, that kills *Hafnia* sp. JB232, a member of the model community. TS33 is easily propagated in the lab and naturally co-occurs in the cheese community, rendering it a prime candidate for the study of host–phage interactions. We performed growth assays of the *Hafnia*, TS33, and the fungal community members, *Geotrichum candidum* and *Penicillium camemberti*. Employing Random Barcode Transposon Sequencing experiments, we identified candidate host factors that contribute to TS33 infectivity, many of which are homologs of bacterial O-antigen genes. *Hafnia* mutants in these genes exhibit decreased susceptibility to phage infection, but experience negative fitness effects in the presence of the fungi. Therefore, mutations in O-antigen biosynthesis homologs may have antagonistic pleiotropic effects in *Hafnia* that have major consequences for its interactions with the rest of the community. Ongoing and future studies aim to unearth the molecular mechanisms by which the O-antigen of *Hafnia* mediates its interactions with its viral and fungal partners.

## Introduction

Bacteriophages are the most abundant replicating entities on the planet. Despite their small size, they possess unique life cycles that provide myriad opportunities to interact with and profoundly impact their hosts. Bacteriophages and their hosts boast an extraordinary genetic diversity, suggesting that host–phage interactions are multifaceted [1, 2]. The detailed mechanisms of several host–phage interactions have been extensively documented, revealing their robust impacts on host biology. The ecological interactions between bacteriophages and their bacterial hosts occur within the context of a microbial community (microbiome), where phages have significant impacts on host abundance, gene expression, diversification, and evolvability [3–6]. In fact, bacterial processes such as quorum sensing, biofilm formation, and metabolism are affected by host–phage interactions [7–9].

Despite our extensive knowledge on the molecular outcomes of host–phage interactions, current understanding of the impact of community context on these interactions is quite sparse. Moreover, some of the current literature available on the impacts of ecological contexts on host–phage interactions is contradicting [10]. Some studies show that the community may constrain host–phage interactions [11], while other studies show the positive effect of their presence in the microbial community [12, 13].

Similarly, there are no generalized conclusions drawn about the role of community context on host–phage coevolution. In some ecosystems, community presence constrains host–phage coevolutionary dynamics. In one study, the community was shown to inhibit fluctuating selection dynamics by driving the bacterial host to extinction [11]. This finding is supported by other work wherein phage resistance of the focal bacterial host is stymied by the presence of competing bacteria [12]. Conversely, there are few examples where bacterial resistance to phage infection appears to be driven by community. In one example, community presence encourages the diversification of the phage, resulting in innovations that broaden the host range and enable the phage to navigate evolutionary trade-offs [14, 15]. Furthermore, the cost of resistance by one mechanism (e.g. surface receptor mutation) can be exacerbated by the community to the point where the host favours another mechanism (e.g. CRISPR) [16]. Still, there is evidence that some communities do not affect host–phage coevolution [17].

Time is a limiting factor in these studies as many of these experiments take place over 15 generations on average, which may provide insufficient time to observe long-lasting ecological and evolutionary effects [10]. What is more, the microbes under investigation were cultured in an environment that is quite different from their natural habitat. Growth conditions that vary from those in which the microbe has evolved can directly and profoundly impact bacterial and phage growth patterns, thereby potentially altering outcomes. By designing experiments that occur over a realistic ecological and evolutionary timeline, and that closely mimic the fundamental niche of the microbes, we can paint a clearer picture of the rich biology that takes place within microbiomes.

In this study, we employ a model community of cheese microbes to investigate the interactions between phages, their hosts, and the wider microbial community. Cheese surface biofilms, or rinds, are taxonomically diverse (containing fungi, bacteria, and phage) microbial communities that follow reproducible growth patterns [18]. Moreover, the relative simplicity (in terms of few species per major taxonomic group) of cheese-associated microbial communities renders them easily manipulable in the laboratory and enables us to completely recapitulate them community *in vitro*. Of cheese rind microbiomes, the bloomy rind microbiomes, such as found on Brie cheese, represent the simplest communities [18]. An experimental model of this microbiome representing the majority of the community alpha diversity was developed previously to study the genetic basis of interspecies interactions [19]. The model Brie community contains three species—the bacterium *Hafnia* sp. JB232 (hereafter *Hafnia*), the yeast *Geotrichum candidum,* and the mould *Penicillium camemberti*. However, to date, phage has not been included in experimental models of cheese microbiomes.

For the present study, we expand the representative Brie community by introducing a novel *Hafnia*-infecting bacteriophage, TS33, isolated from the same type of cheese as the original model community isolates. In effect, we have developed a model host–phage interaction within the already existing model community that has allowed us to study how host–phage interactions occur within a community context. Our primary approach involves using a high-throughput genetic screen that employs barcoded transposon mutant libraries (RB-TnSeq) to (i) discover novel microbial interactions within a community context and (ii) investigate how increasing community complexity affects these interactions. TnSeq methods were originally developed to determine the genetic basis of specific phenotypes. Comparisons of gene frequency under various conditions allow us to highlight key genes that promote and hinder growth [20]. Using a newly generated barcoded transposon insertion library in *Hafnia*, we investigate the effects of a community of fungal partners, *P. camemberti* and *G. candidum*, on the ecology and evolution of the *Hafnia*–phage interaction. This work illustrates the impact of the ecological context on the molecular outcomes of interactions between bacteria and their phages and the role of bacterial cell surface structures in mediating these interactions. Further, our results highlight the importance of working within a community context to fully understand the evolutionary pressures that influence phage–host interactions and coevolution.

## Materials and Methods

### Media preparation

Community growth assays were performed using 10% cheese curd agar (CCA) at pH 7 (10% freeze-dried cheese curd (Jasper Hill Farm, VT), 3% NaCl, 0.5% xanthan gum, and 1.7% agar); 10 M NaOH was used to buffer the pH from 5.5 to 7 [18]. All growth assays and inoculations were performed at room temperature.

### Bacterial, fungal, and phage strain selection and preparation

All bacterial and fungal isolates were obtained from a natural rind cheese as previously described [18]. The community was selected based on its success as a model in a prior study conducted in the group [19].

As *Hafnia* is the only bacterium in the model community, it was used to isolate phages from a different batch of the bloomy rind cheese from which it was originally isolated. The cheese rind was scraped and homogenized in SM buffer (100 mM NaCl, 8 mM MgSO_4_, 50 mM Tris-Cl). The suspension was vortexed vigorously and centrifuged at 13 000 rpm, at 4°C. The supernatant was filtered using a 0.45 μM filter. The filtrate was then serially diluted; each dilution was mixed with 200 μl of a late log culture of *Hafnia* and 4.5 ml soft agar (0.05% Bacto-agar, 25% Luria-Bertani (LB) agar) and poured onto solid LB media. After 24 h, the resulting plaques were picked and struck out three times on solid LB medium with a soft agar lawn of *Hafnia*. On the third quadrant streak, one plaque was picked and cultured in liquid LB medium with a colony of *Hafnia*. After 16–18 h of incubation, the cells were pelleted and the supernatant was filtered using a 0.45 μM filter. This phage lysate was stored at −80°C with fresh 1XPBS-40% glycerol.

To prepare stocks of *P. camemberti* (Danisco—CHOOZIT, strain PC SAM 3 LYO 10D) and *G. candidum* (Danisco—CHOOZIT, strain GEO13 LYO 2D), spores were obtained from Danisco and resuspended in 1XPBS-Tween0.05%. Spore stocks of equal volume were aliquoted with fresh 1XPBS-40% glycerol and stored at −80°C. To quantify the stocks, aliquots were frozen for 48 h, after which they were thawed and plated on solid LB medium. For each fungal strain, we calculated the number of cfus per millilitre of strain stock.

### Phage TS33 genome sequencing and phylogeny

As previously described, material from TS33 glycerol stocks was cocultured with a pure colony of *Hafnia* overnight. The suspension was vortexed vigorously and centrifuged at 13 000 rpm, at 4°C. The supernatant was filtered using a 0.45 μM filter and treated with 10% volume of pure chloroform to kill remaining bacterial cells. The suspension was mixed gently for 30 min at room temperature and centrifuged for 10 min at 13 000 rpm. The aqueous phase was removed and treated with GE Nuclease Mix, followed by Ethylenediaminetetraacetic acid (EDTA), Proteinase K, and 10% sodium dodecyl sulfate (SDS). A phenol-chloroform extraction was performed on the retrieved phage DNA. The DNA was sequenced using an iSeq 100 (Illumina). One hundred sixty-eight thousand sequencing reads were obtained. These reads were assembled into a single contig using SPAdes (version 3.13.0) [21], which was uploaded to the VipTree server for phylogenetic analysis [22]. The TS33 genome is available in GenBank under the Accession Number OR844384.

### Transposon mutant library construction in *Hafnia*

*Hafnia* was mutagenized by conjugation with *E. coli* strain APA766 (donor WM3064, which carries the kanamycin-resistant pKMW7 Tn5 vector library containing 20 bp barcodes) [23]. In pilot experiments, improved conjugation efficiencies were observed when the donor and recipient strains were at the mid-log phase prior to conjugation. The APA766 donor strain was grown in LB-kanamycin:diaminopimelic acid (DAP) (1 ml frozen stock into 99 ml of LB with 50 μg/ml kanamycin and 60 μg/ml DAP) at 37°C at 200 rpm until the culture reached the mid-log phase. *Hafnia* was grown in LB at 30°C at 200 rpm until the culture reached the mid-log phase. *Escherichia coli* donor cells were pelleted and washed twice with 100 ml of LB without antibiotics. Donor and recipient cells were mixed at a 1:1 cell ratio based on OD600 measurements, pelleted, and resuspended in 100 μl. Forty microlitres of the mix was plated on a nitrocellulose filter on an LB plate containing 60 μg/ml DAP. Conjugation took place at 30°C overnight. Eight conjugations were performed. The conjugations were each resuspended in 2 ml of LB broth, and then, 100 μl was plated on an LB:kanamycin (50 μg/ml) agar plate (containing no DAP). This was done for a total of 120 selection plates. Plates were left at room temperature until single colonies formed (~3 days). Given that wild-type *Hafnia* is not resistant to kanamycin, and the *E. coli* donor library is a DAP auxotroph, only *Hafnia* with transposon insertions should have formed single colonies on these plates. The resulting transconjugants were scraped into LB from the selection plates and pooled together. The resulting pool was mixed well and then diluted back to an OD600 of 0.2 in liquid LB:kanamycin (50 μg/ml). The pool was then grown to mid-log phase, and two 5 ml samples were taken and pelleted. Cell pellets were frozen at −80°C for later library characterization. Sterile glycerol was then added to a final concentration of 15%. One millilitre glycerol stocks were stored at −80°C. The final library was estimated to contain ~160 000 mutants. This estimate was achieved based on counting the number of individual colonies that grew on a few of the selection plates after conjugation and then multiplying by the total number of plates harvested for the library. Colony distribution across plates was fairly even.

### Library preparation and sequencing

Genomic DNA was extracted from the cell pellet of the *Hafnia* mutant library using phenol:chloroform:isoamyl alcohol (ph 8) [18]. Library preparation was conducted by the standard ≥100 ng protocol from the NEBNext Ultra II FS DNA Library Prep Kit for Illumina (NEB) with minor modifications. Five hundred nanograms of input DNA and a 7-min fragmentation incubation were used. For adaptor ligation, a custom splinkerette adaptor prepared at 15 μM was used (duplexed DNA of /5Phos/GATCGGAAGAGCttttttttttcaaaaaaaa/GTGACTGGAGTTCAGACGTGTGCTCTTCCGATC*T). The USER enzyme step was not performed. For size selection, 0.15X (by volume) NEBNext Sample Purification Beads (NEB) were used for the first and second bead selection steps. Before enrichment, DNA was digested with BsrBI (NEB) for 20 min at 37°C prior to heat inactivation at 80°C for 20 min. The DNA was then purified using 1X AMPure XP beads (Beckman Coulter) in preparation for polymerase chain reaction (PCR) enrichment. The NEBNext Ultra II FS DNA Library Prep Kit for Illumina (NEB) PCR protocol was used, with custom primers and 30 cycles for the PCR step (P6_ET-Seq2_3_R1: AATGATACGGCGACCACCGAGATCTACACGTCGTCacacTCTTTCCCTACACGACGCTCTTCCGATCTNNNNNNGATGTCCACGAGGTCTC*T and P8_ET-Seq3_R2: CAAGCAGAAGACGGCATACGAGATACATCGGTGACTGGAGTTCAGACGTGT*G). Following library preparation, PE150 sequencing was done by Novogene using a Novaseq 6000 platform (Illumina). 15 GB of sequencing data was received. The library sequencing file is publicly available at NCBI under SRA Accession Number SRX22606296, or BioProject Accession Number PRJNA1043638.

### Library characterization

Sequencing reads were analysed using the Perl script MapTnSeq.pl from Wetmore *et al.* [23]. This script maps reads to the *Hafnia* genome and is publicly available at https://bitbucket.org/berkeleylab/feba. The script DesignRandomPool.pl was used to generate the file of mapped barcodes. There was a total of 165 694 insertions mapped, with 103 169 located within the central 10%–90% of a gene, which is the standard for Tn-Seq experiments. Given that ~85% of a bacterial genome is protein-coding, and that the transposon insertions are totally random in this experiment, we would expect 140 839 insertions would be in genes. Ignoring the first and last 10% of genes gives an expected ~112 000 mutants (80% of 140 839), suggesting a reasonable number of mutants obtained for this study. There were central insertions in 88% of protein-coding genes.

### *In vitro* community density experiment using RB-TnSeq library in *Hafnia*

#### Library preculture

The *Hafnia* transposon library was thawed on ice and cultured in liquid LB with kanamycin (1 μg/ml) to the mid-log phase. Five millilitres of the preculture was washed with PBS-Tween, pelleted, and stored at −80°C to be used as the T0 reference in the fitness analysis.

#### Inoculations

The remaining *Hafnia* cells, as well as thawed spores of both fungi were pelleted and washed separately in 1XPBS/Tween before inoculation. Respective to the condition of growth, 2.4 × 10^5^ cfus of the precultured *Hafnia* library, 3.37 × 10^5^ cfus *P. camemberti* (thawed from the frozen stock), 3.59 × 10^5^ cfus *G. candidum* (thawed from the frozen stock), and 4.18 × 10^3^ pfus TS33 (filter-sterilized from a coculture of *Hafnia* and TS33) were inoculated on cheese curd agar (CCA) plates using sterile glass beads. To represent the 3 days of the experiment, we inoculated three sets of plates, each containing three biological replicates (made from three different library stocks) for each growth condition, and harvested each set of plates on a separate day.

#### Harvest

At *T* = 24, 48, and 72 h, cells, spores, and phage particles were harvested from the fitness assays. For each harvest, CCA plates were flooded with 2–3 ml of 1XPBS-Tween0.05% and cells were gently scraped off. Cells and/or spores were pelleted, and the pellets were washed twice. In conditions containing phage, the supernatants generated after each wash were combined. We made three stocks for each biological replicate to obtain three technical replicates for each. Before quantification, cells and supernatants from each fitness assay were aliquoted and stored in 1XPBS-40% glycerol at −80°C before quantification. The remainder was stored at −80°C without glycerol for DNA extraction.

#### Growth quantification

Cells/spores in frozen stocks were pelleted by centrifugation and washed with 1XPBS-Tween0.05%. The cells were resuspended in 200 μl 1XPBS-Tween0.05%, serially diluted, and spread evenly on solid LB media using glass beads. Frozen aliquots of phage were thawed, serially diluted, and titred on a soft agar lawn of *Hafnia* (as described above). The number of colonies and plaques were used to calculate the number of colony-forming units (cfus) and plaque-forming units (pfus), respectively, in 1 ml of harvested material. The bacterial and fungal colonies were distinguished by their colony morphologies (*Hafnia* sp. JB232 forms smooth, translucent colonies, *G. candidum* forms white, flat colonies that form a web-like pattern, *P. camemberti* forms white, puffy, and raised colonies). *Penicillium camemberti* does not sporulate efficiently and thus could not be measured accurately using these plating-based methods. Subsequently, we calculated the total number of cfus and pfus harvested. For each biological replicate, we used the mean cfus/pfus of three technical replicates. These values were analyzed for statistical significance using Tukey’s multiple comparisons of means. Quantile–quantile plots were used to test for normality using R version 4.2.2.

#### Genomic DNA (gDNA) extraction

From each fitness assay, gDNA was extracted using phenol:chloroform:isoamyl alcohol after 3 days (ph 8). Cell lysis was conducted by adding a homemade lysis buffer (10 mM Tris-Cl pH 8, 100 mM EDTA pH 8, 1% SDS, 10 μg/ml RNAse A, 1 mg/ml lysozyme) to the pellet, vortexing the tubes at maximum speed and incubating at 37°C for 1 h. An equal volume of phenol:chloroform:isoamyl alcohol was added to the lysate, after which the tubes were centrifuged at maximum speed for 15 min at 4°C. To precipitate the gDNA, 0.1 volume of 10 M ammonium acetate and an equal volume of extremely cold isopropanol was added to the aqueous phase (upper layer). After centrifuging for 3 min at maximum speed, the pellet was washed with fresh 70% ethanol and resuspended in 25 μl of DNAse/RNAse-free water overnight.

#### Barcode amplification and sequencing

After resuspension, the DNA was quantified via Qubit dsDNA HS assay kit (Invitrogen). Subsequently, we used the BarSeq PCR protocol devised by Wetmore *et al.* to amplify only the barcoded region of the transposons [23]. The PCR reaction and program used were also previously reported by Morin *et al.* [19]. In total, we performed 13 PCRs (T0 sample and 12 harvest samples) involving 13 different multiplexing indexes. Ten microlitres of each PCR product was pooled, after which the entire pool was purified using the Qiagen MinElute purification kit and quantified via Qubit dsDNA HS assay kit (Invitrogen) and sequenced at Novogene on 1 HiSeqX PE150 lane (6 bp, i7 single index, Illumina). The output of the lane was 375 million reads. These sequences are publicly available at NCBI under SRA Accession Numbers SRX22606139 through SRX22606151 or BioProject Accession Number PRJNA1043638.

#### RB-TnSeq data processing and fitness analysis

To calculate fitness values for each gene represented in the library, the demultiplexed barcode reads were processed using the RB-TnSeq data analysis pipeline assembled by Wetmore *et al.* [23]. Each script is available to the public at https://bitbucket.org/berkeleylab/feba. The pipeline quantifies the number of reads associated with each barcode to determine the abundance of each insertion mutant under each condition, at *T* = 0 and *T* = 3. Reads that do not map to the characterized barcodes in the library are filtered out, as well as barcodes that are counted less than three times in the T0 sample. Moreover, genes represented by less than 30 barcode reads are excluded from further analysis. For the barcodes that meet these parameters, a raw fitness value (log2 change in abundance over 3 days) is calculated and assigned. The weighted average of the fitness values of all insertion mutants in a single gene is calculated as the raw fitness value in that gene. The raw fitness value of each gene is subsequently normalized in two stages. First, the smoothed median is subtracted from each fitness value. This calculation is essential to account for variance in copy number as genes closer to the replication fork have a higher copy number. Second, we assume that most insertions have neutral fitness effects or fitness effects of 0. To this end, the mode of the fitness distribution is subtracted from each fitness value (for more details, see Wetmore *et al.* [23]).

Because we assume that most genes have neutral fitness, our analysis aims only to highlight genes with strong fitness effects, that is, genes whose fitness values were reliably different from 0. A moderate *t*-statistic (*t*-score) was calculated for each gene as the ratio of the gene fitness to its standard deviation (for more details, see Wetmore *et al.* [23]). T-scores were further used to filter genes with neutral fitness from the analysis. Specifically, we assumed that most genes with reliable neutral fitness had a *t*-score less than 3 and thus were excluded.

For the remaining genes, we calculated an average fitness score and *t*-score for the three biological replicates in each condition. Genes were denoted as having strong positive fitness effects if their abundance of these genes at the end of the experiment exceeds their abundance in the inoculum, as well as the abundance of all other mutants in the library under the growth condition. These genes also had a fitness value that was >0. In contrast, negative fitness effects are observed in genes whose insertion mutants exhibit a strong growth deficit after 3 days of growth.

#### Selection and isolation of TS33-resistant mutants for fitness assay

TS33-resistant mutants were isolated from the RB-TnSeq library in two ways.

First, the library was precultured to the late-log phase in liquid LB medium with kanamycin (1 μg/ml) and 1.5 × 10^8^ pfus TS33. The cells were pelleted, washed twice, and resuspended in 1XPBS-Tween0.05%. The cells were then diluted, and each dilution was plated on solid LB media using sterile glass beads. Eight colonies were picked and struck twice on solid LB media. Titre assays of TS33 on soft agar lawns on these strains showed that they are completely resistant to TS33 infection. Whole genome sequencing was conducted using a NextSeq 2000 at SeqCenter, LLC (Illumina). Two *wzy* mutants were selected from this set.

Second, the library was precultured to the late-log phase in liquid LB medium with 1.5 × 10^8^ pfus TS33 and no kanamycin. The cells were pelleted, washed twice, and resuspended in 1XPBS-Tween0.05%. The cells were then diluted, and each dilution was plated on solid LB media using sterile glass beads. forty-two colonies were picked and struck twice on solid LB media. Titre assays of TS33 on soft agar lawns on these strains showed varying levels of resistance across the mutants, with some being completely resistant and others showing significantly decreased susceptibility to TS33 infection. Barcode amplification was conducted as previously described (see above). Whole genome sequencing was conducted using on a NextSeq 2000 at SeqCenter, LLC on two mutants in the genes *manC* and *rfaH* (mutant 9 and mutant 44 on Supplementary Table 1, respectively) (Illumina). We do not have a whole-genome sequence for the *rfaL* mutant.

For each mutant, we performed single plaque assays and used the results to calculate the Efficiency of plaquing of TS33 as the ratio of the number of pfus of TS33 on lawns of each mutant to the number of pfus produced on a lawn of the wild type (Supplementary Table 1).

### *In vitro* growth assay using insertion mutants in *Hafnia*

#### Inoculation

The five selected mutant strains and the wild-type *Hafnia* were precultured (from colonies picked from quadrant streak plate) to late-log phase in liquid LB medium. The cells were pelleted by centrifugation and washed twice with and resuspended in 1XPBS-Tween0.05%. Frozen stocks of *G. candidum* and *P. camemberti* were washed twice with and resuspended in 1XPBS-Tween0.05%. For each strain, ~1.08 × 10^8^ cfus on average were plated on CCA medium, with or without both fungi (3.82 × 10^6^ cfus of *G. candidum* and 9.82 × 10^5^ cfus *P. camemberti* were plated). We used three biological replicates (three different overnight cultures of *Hafnia*) for each condition.

#### Harvest

After 72 h, the cells and spores were harvested using 1XPBS-Tween0.05% as previously described.

#### Quantification of growth

The harvested cells and spores were diluted in 1XPBS-Tween0.05% and plated on solid LB medium. *Hafnia* colonies were counted and the number of cfus harvested was calculated for each mutant under each condition of growth (alone or with fungi). We made three viable count plates for each biological replicate to obtain three technical replicates for each. We calculated the efficiency of plaquing (EOP; as previously described) of TS33 on the mutant and wild-type lawns. These values were analyzed for statistical significance using the Tukey test in R, and quantile–quantile plots were used to test for normality.

## Results

### Isolating a lytic bacteriophage that kills *Hafnia*

To establish a model system for studying host–phage interactions within our Brie community, we first isolated a bacteriophage that infects the bacterial member of this community, *Hafnia* sp. JB232. This bacterium was first isolated from a bloomy rind cheese in 2011 and is believed to be a novel species of *Hafnia* (Fig. S1A, [18, 24, 25]). Using this bacterium as a host, we isolated bacteriophage TS33 from a batch of the same bloomy rind cheese that was made in 2019.

**Figure 1:**
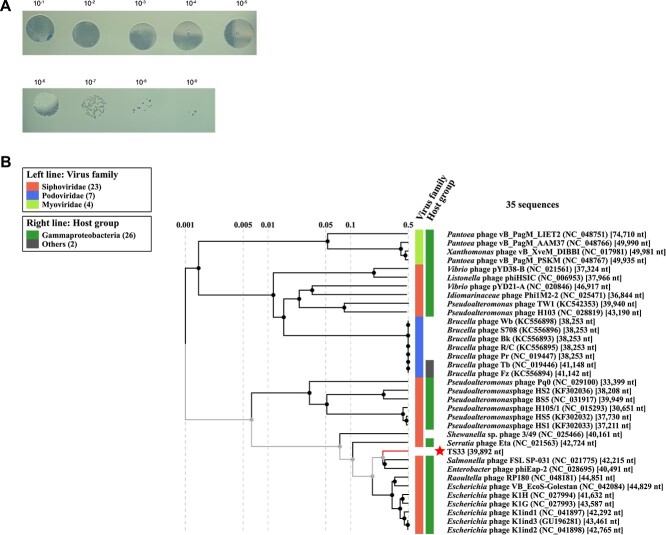
Novel lytic *Hafnia*-infecting bacteriophage TS33 is phylogenetically related to members of the virus family *Siphoviridae*. (A) Plaque assay of phage TS33 on *Hafnia* lawn. Three microlitres of serially diluted suspensions of pure phage lysates were spotted onto a *Hafnia* lawn. (B) Proteomic tree showing the predicted phylogenetic classification of TS33 among *Siphoviridae*. Phylogeny was determined based on whole genome comparison of the TS33 proteome to the proteomes of well-characterized bacteriophages using the Viral Proteomic Tree (ViPTree) Server.

Plaque assays revealed that TS33 uses the lytic cycle to infect *Hafnia* and produces a titre of more than 10^10^ pfu/ml following overnight incubation with the host (Fig. 1A). The phage TS33 genome was sequenced and subsequently analyzed using the Viral Proteomic Tree (VipTree) server to determine genome-wide similarities to a bacteriophage reference database [22]. These whole genome comparisons predict that phage TS33 is a member of the viral family *Siphoviridae*. Phage TS33 is most closely related to *Salmonella* phage FSL SP-031 and *Enterobacter* phage phiEap-2, the latter of which infects multidrug-resistant *Enterobacter aerogenes* (Fig. 1B, [26]).

### Identifying genetic requirements of *Hafnia* growing under different conditions

We used a high-throughput genetic approach to determine how different ecological contexts affect the genetic requirements of *Hafnia*. An RB-TnSeq library was developed in *Hafnia* by mating with the *Escherichia coli* Keio_ML9 RB-TnSeq library from Wetmore *et al.* [23]. The *Hafnia* library contains 103 169 insertion mutations (and thus mutants) in 58 869 distinct locations. Around 88% of the protein-coding genes are disrupted, when considering insertions being in only 10%–90% region of those genes (Fig. S1B, [27]). Moreover, for each gene represented in the library, there were on average 25.6 different insertion mutants generated (based on the insertion location within the gene).

**Figure 2:**
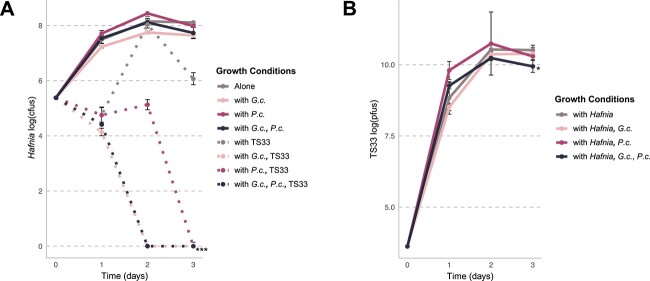
*Hafnia* growth is inhibited in the presence of a community of fungi and bacteriophage TS33. The *Hafnia* library was grown alone, and with phage and/or fungi on *in vitro* cheese medium for 3 days. The harvested cells (A) and phage particles (B) were plated on each day and quantified using the total number of cfus and pfus, respectively. Asterisks indicate a statistically significant (Tukey’s multiple comparison of means, *P* < .05) decrease in the total growth of *Hafnia* RB-TnSeq library under different growth conditions compared to growth alone. The number of asterisks represents the degree of statistical significance (* = *P* < .05, ** = *P* < .01, *** = *P* < .001). Error bars indicate standard deviation.

To determine the effect of ecological context on the genes required for *Hafnia* growth, the pooled transposon mutant library was inoculated on an *in vitro* cheese medium either alone or in the presence of the fungal partners from the Brie community, *G. candidum* and *P. camemberti*, in a 1:1 ratio (Fig. 2, Fig. S3). Additionally, each experimental condition was performed with or without phage TS33 (multiplicity of infection (MOI) = 0.001). On each day of the experiment, community samples were harvested, diluted, and plated for counting (Fig. 2).

*Geotrichum candidum* grows steadily over 3 days, attaining levels that do not appear to be impacted by the presence of bacteria and phage, bacteria only, or *P. camemberti* (Fig. S3A). Similarly, *Hafnia* growth levels are not significantly impacted by the presence of either fungus or both fungi (Fig. 2A, Fig. S2A). However, in the presence of the phage, the number of *Hafnia* cfus decrease on Day 1, recover on Day 2, and decline again on Day 3. Moreover, *Hafnia* growth conditions that include phage TS33 and any combination of the fungi exhibit complete extinction of viable *Hafnia* cells from the community by Day 3 (Fig. 2A; Tukey test, *P <* .05). These data suggest that when both phage and fungi are present, *Hafnia* fails to recover its population levels. Moreover, our data show that phage TS33 density does not change in three of the four growth conditions, except the one containing both *G. candidum* and *P. camemberti*. We observe that the presence of both fungi significantly decreases the number of viable phage particles (Tukey test, *P <* .05) after three days of growth (Fig. 2B, Fig. S2B). The possibility exists that the fungus-led extinction of host cells resulted in a decrease in the number of TS33 virions. However, this remains puzzling since the decrease in TS33 over the 3 days is far less than what we observe in the host cells, suggesting that TS33 virions somehow maintain stability outside of the host for an extended period of time. Nevertheless, our results indicate that the presence of the community negatively affects the growth of both *Hafnia* and phage TS33.

**Figure 3:**
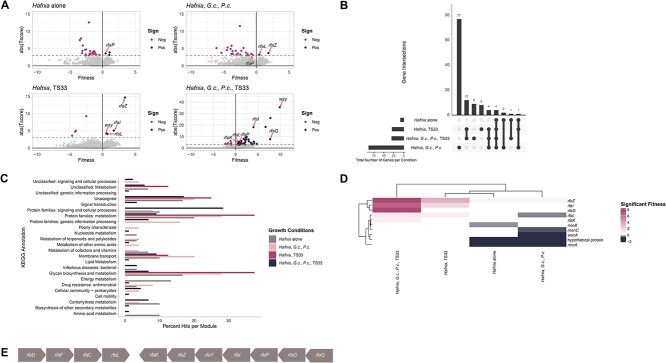
Mutations in genes controlling O-antigen synthesis and ligation may cause pleiotropic effects in *Hafnia*. (A) The barcodes of the *Hafnia* library plated under various growth conditions were amplified and sequenced. The original RB-TnSeq bioinformatic pipeline developed by Wetmore *et al.* [23] was used to quantify each barcode and determine the abundance of each mutant in each gene represented in the library. The sum of the abundances of each mutant for each gene was used to assign a fitness value to each gene. Mutations with a significant negative and positive fitness effect are highlighted. (B) The number of genes unique to and shared by the four growth conditions were determined using the UpSetR package developed by Lex *et al.* [28] (updated by Conway *et al.* [29]). (C) The KEGG Mapper was used to identify gene pathways enriched under each condition. (D) ComplexHeatMap in R was used to compare the enriched genes under each condition [30,31]. (E) Schematic representation of *rfa* operon in *Hafnia* wild type. Adapted from Pagnout *et al.* [32].

To determine the relative fitness of the *Hafnia* mutants in the library in each growth condition, we compared the abundance of each mutant in each growth condition after 3 days of growth to its abundance at the start of the experiment. DNA was extracted from the cells in the inoculum and cells harvested on Day 3. RB-TnSeq barcodes therein were amplified using PCR and sequenced. Using sequence data, we calculated the abundances of each barcode and therefore of each insertion mutant at both timepoints using the standard fitness pipeline [23]. For each condition, a fitness value was assigned to each insertion mutant by calculating the log2 change in barcode abundance over 3 days (Fig. 3A, Fig. S4B, [28, 29]). Next, we assigned a raw fitness value to each gene represented in the library by calculating the weighted average of the fitness values assigned to all insertion mutants in that gene. The raw fitness for each gene was then normalized in two steps: (i) first, by chromosomal location to account for variance in copy number and (ii) second, by assigning neutral fitness of 0 to most genes (for more details, see Materials and Methods and [23]).

Our analysis only included genes whose fitness values were significantly different from 0, as these genes exhibit a significant effect on strain fitness. To this end, a moderated *t*-statistic (*t*-score) was calculated for each gene. In every condition, we assumed that most genes with an absolute *t*-score ≥3 had a fitness value that was reliably different from 0 [19]. Thus, our analysis excluded genes that did not meet this criterion. Fitness effects that are >0 represent positive fitness effects; the abundance of these genes at *T* = 3 exceeds their abundance at *T* = 0, as well as the abundance of all other mutants in the library under the growth condition. Conversely, the mutants who decrease in abundance through the experiment experience a negative fitness effect.

Of the 4021 protein-coding genes represented in the library, we successfully obtained fitness values and moderate *t*-statistics for 3863 (96.07%). We employed *t*-score statistics to filter out genes that lacked a strong fitness effect. In our dataset, this was ~96% of all genes (3713/3863). There were 150 genes with strong fitness effects in at least one condition, and 4 of these were conserved across all 4 conditions (Fig. 3B, [28, 29]).

Next, we compared the gene functions assigned to these 150 genes among the different ecological contexts of *Hafnia* growth to identify whether certain molecular pathways are overrepresented therein. To this end, each gene with a strong fitness effect was annotated using the KEGG Orthology and Links Annotation (KOALA) BLAST and mapped to the KEGG BRITE database (Fig. 3C) [33]. For each condition, we calculated the percent of the total annotations represented by each KEGG annotation and then looked specifically for groups that were abundant in all four conditions of growth. Genes classified under “Protein families: metabolism” and “Glycan biosynthesis and metabolism” were present in all four conditions. It is important to state that 52.9% of the genes classified under “Protein families: metabolism” received the annotation “Glycan biosynthesis and metabolism” or the subannotation “Lipopolysaccharide biosynthesis proteins.” Moreover, the glycan biosynthesis and metabolism genes are all involved in lipopolysaccharide (LPS) biosynthesis. The LPS is a glycolipid that is comprised of three parts—lipid A, the core polysaccharide, and the O-antigen, which is the most outward-facing part of the structure. The LPS is well documented as a critical cell structure for combatting attacks from bacteriophages, other bacteria, and chemicals [34,35]. Specifically, it is a common adsorption site for diverse bacteriophages [36,37]. Here, we see that the LPS genes are present in some way under all conditions of growth.

To determine the specific impacts of these genes in each condition, we investigated the fitness effects of each gene associated with glycan/LPS biosynthesis and metabolism. The LPS biosynthesis genes were primarily annotated as *rfa* genes (known players in LPS biosynthesis in *E. coli*, [38]) and glycosyltransferases predicted to be involved in cell wall biosynthesis (Fig. 3E, adapted from [32]).

Insertions in the *rfaL* gene, which synthesizes the O-antigen ligase, exhibit positive fitness effects in all conditions containing phage, and negative fitness effects in the presence of fungi only (Fig. 3A, Fig. 3D, [30,31]). These fitness effects were stronger than those observed when *Hafnia* grows alone. Furthermore, disruption of the *rfaZ* (KDO-transferase) gene has a relatively small positive effect on the fitness of *Hafnia* growing with fungi; however, this effect is three times greater when *Hafnia* grows with phage TS33 and is seven times greater when *Hafnia* grows in the community (Fig. 3A and D). Another operon encoding predicted glycosyltransferases that contribute to cell wall biosynthesis follows a similar pattern; overall, we observe that mutations in any genes from this operon have negative fitness effects on *Hafnia* growing with fungi and positive fitness effects during growth with phage or the whole community. In other words, in the presence of TS33, disrupting LPS biosynthesis appears to provide a fitness benefit to *Hafnia* but may offer a fitness cost in the presence of the fungi.

The positive fitness effects that we observe in these genes when *Hafnia* is growing with the community may be attributed to strong selection for insertions in these genes by the phage. Subsequently, they succumb to potentially negative interactions with the fungi, evidenced by the stark decrease in the growth of *Hafnia* in the community context compared to all other conditions. Thus, we hypothesized that some genes involved in LPS synthesis might have pleiotropic effects in *Hafnia*.

### Determining the role of LPS in host–virus–fungus interactions.

We hypothesized that the phage-susceptible insertion mutants experience high levels of phage predation, resulting in the community being dominated by TS33-resistant mutants and accounting for the spike in *Hafnia* counts on Day 2. However, eventually, the *Hafnia* counts recover, due to the emergence of counter-resistant mutations in phage TS33 and evolutionary rescue [39]. However, in the presence of either fungus or both fungi, the phage TS33-resistant mutants experience negative fitness, resulting in their decrease in growth in any ecological context containing fungi (Fig. 2A).

To directly test this hypothesis, we investigated whether phage TS33-resistant mutants with disrupted LPS genes experience a fitness deficit in the presence of both fungi. We subjected the *Hafnia* library to high titres of phage TS33 for 24 h in liquid LB. The remaining viable cells were plated on solid LB media, and the emergent colonies were picked and struck three times to purify the strains. Phage TS33 was diluted and titered on top agar lawns of each mutant. In total, we isolated and sequenced the barcodes of 50 mutants, each displaying decreased or no susceptibility to the TS33 lysate compared to the wild type (Supplementary Table 1).

**Figure 4:**
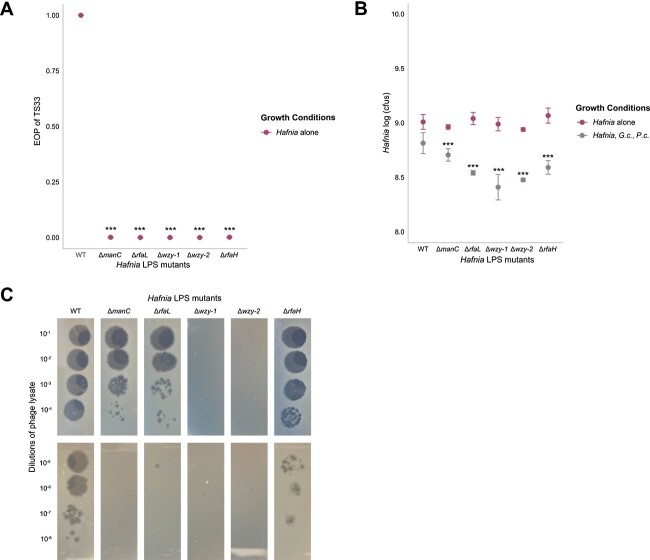
LPS mutations increase *Hafnia* resistance to TS33 infection but decrease bacterial growth in the presence of the fungi. (A) Comparing EOP of TS33 in wild type to its EOPs in mutant *Hafnia* strains. EOP is the ratio of the number of TS33 phage particles (shown here as the number of pfus) produced on a soft agar lawn of each bacterial strain by the number of pfus produced following infection on a soft agar lawn of the wild type. Asterisks indicate a statistically significant (Tukey’s multiple comparison of means, *P <* .05; (* = *P <* .05, ** = *P <* .01, *** = *P <* .001) decrease in the EOP of TS33 on mutant *Hafnia* strains compared to the wild type. (B) Effects of fungi on growth of *Hafnia* strains over 3 days, measured in the number of viable bacterial cells (represented here in cfus). Asterisks indicate a statistically significant (Tukey’s multiple comparison of means, *P <* .05) decrease in the total growth of *Hafnia* strains in the presence of *G. candidum* and *P. camemberti*, compared to growth alone. Error bars indicate standard deviation. (C) Images of phage plaquing on various *Hafnia* mutants.

Sanger sequencing of the mutant barcodes revealed disruptions in the genes associated with biosynthesis of the cell wall, especially of the LPS. From the 50 mutants, we selected 5 that were represented in our RB-TnSeq results in at least one of the three experimental conditions: one in *manC* (*cpsB* in *E. coli*, involved in the synthesis of the growing O-antigen [40]), one in *rfaL* (O-antigen ligase), one in *rfaH* (transcriptional regulator *rfa* (LPS-synthesizing) gene expression [41]), and two in *wzy*, which encodes an O-antigen polymerase (Fig. 4A, Fig. S2C, [42]). The mutant and wild-type *Hafnia* cells were then inoculated on *in vitro* cheese medium with or without the fungal community members. After 3 days, the cells were harvested and quantified. For each insertion mutant plated, we compared the number of cfus after growth with fungi to that after growth alone (Fig. 4B, Fig. S2D).

The *wzy* mutants were completely resistant to infection by phage TS33, whereas the *manC*, *rfaL*, and *rfaH* mutants exhibited statistically significant reductions in their susceptibility. This reduction in growth is not a result of insertion-mediated growth deficits, since the total growth of WT alone is not statistically different from the total growth of mutants alone (Fig. 4B; Tukey test, *P* > .05). From these results, we can observe a fitness benefit to the *Hafnia* cells by the disruption of genes involved in O-antigen synthesis, with the strongest benefit coming from the interruption of the O-antigen polymerase gene (Fig. 4A). In the presence of the fungi, each mutant experienced a significant decrease in growth, compared to the wild type (Fig. 4B, Tukey test, *P <* .05), suggesting a role played by the O-antigen in the modulation of interactions between *Hafnia* and its viral and fungal environment.

## Discussion

Most studies of bacteria–phage interactions completely exclude the natural community wherein the microbes exist naturally. In this study, we used *Hafnia* interactions with phage TS33 as a model to probe the impact of community context on host–phage interactions. Under each condition of growth, we quantified the number of bacterial cells to understand the effects of different combinations of community members on *Hafnia* growth. Moreover, using a high-throughput genetic screen, we were able to further investigate these growth effects by studying the fitness landscape of the *Hafnia* cells in each growth condition. Our analyses reveal that phage can lead to fluctuations in *Hafnia* growth that the bacteria eventually recover from. During the length of our experiment, neither the phage nor fungi alone elicited as drastic a depletion in the final counts of *Hafnia*, as both of them did together. In other words, neither group of community partners is sufficient to produce a strong negative outcome on *Hafnia* growth; both are required for extinction of *Hafnia*.

Similar and contradicting observations have been made by other scientists. In a study of wastewater microbial ecology, Johnke *et al.* unearthed suppressive effects of a microbial community on *Klebsiella* sp. [11]. Under phage-selective pressures, the bacterium experiences more severe impacts on its growth. In the same study, they also show that the presence of some other specific community members contributes to the sustained growth of *Klebsiella*. Conversely, *P. fluorescens* and *P. aeruginosa* both benefitted from a community presence amidst phage predation [12, 13]. Although our results most closely support those projected by Johnke *et al.*, it is probable that the community effects are dependent on community composition. Our studies involved the use of fungi, *Penicillium camemberti* and *G. candidum*, which are known to have negative interactions with other microbes. *Penicillium camemberti* produces effects on *E. coli* that are similar to those exhibited by a beta-lactam antibiotic [43]. Moreover, *G. candidum* is believed to exert oxidative stress on *E. coli* [19]. In this previous study, in the presence of the fungus, *E. coli* depends heavily on genes such as *acrA* and *acrB* that enable it to resist oxidative stress. Moreover, in the presence of the community, these genetic requirements are alleviated, suggesting that activity from one of the community members is either preventing oxidative stress or empowering *E. coli* to deal with it. It is possible that these molecular products of both fungi produce a negative effect on *Hafnia* mutants resistant to phage infection. To further understand the relationship between community presence and growth of focal bacteria, it is essential to investigate the molecular mechanisms that underlie their interactions.

Recently, RB-TnSeq has been applied to the description of host factors required for phage infection. Adler *et al.* outline a study in *Salmonella enterica* serovar Typhimurium where a dense library was generated and subjected to phage predation [44]. They successfully identified over 300 genes required for infection by multiple phages. This led to the identification of cross-resistance conferred by the RpoS and RpoN genes. Similarly, the genetic determinants of phage resistance were also identified in *E. coli* using the same genome-scale approach [45]. Among the genes required for phage infection were those associated with synthesis of the Gram-negative LPS and nutrient transporters. Additionally, a similar method called INSeq was used by Kortight *et al.* to verify candidate receptors for various coliphages, among which LPS and transport genes were identified [46].

Based on our results, we hypothesize that the LPS, specifically the O-antigen, plays a key role in mediating *Hafnia*’s interactions with its community. Specifically, mutations in homologs of O-antigen synthesis (*rfaL*, *wzy*, *manC*) and regulation of the LPS biosynthesis gene expression (*rfaH*) produced a growth deficit in the presence of fungi and increased growth in the presence of phage, compared to the wild type. In future studies, one way to directly test this hypothesis is to investigate the ability of spent, filtered supernatant from *G. candidum* and *P. camemberti* to inhibit the growth of LPS-deficient *Hafnia*. Further, characterizing the O-antigen homologs of *Hafnia* can provide important molecular details about these interactions.

The LPS is a major component of the defence mechanism of Gram-negative bacteria, as it protects them from chemical attacks by other microbes and the environment. The intact LPS is often exploited by phage during attachment to the host [36]. In fact, the O-antigens of some bacteria have been shown to be a specific target of several bacteriophages [47]. Additionally, phages and antimicrobials are known to negatively affect the growth of bacterial cells with compromised LPS [48, 49].

Previous work in the field has demonstrated that LPS modification has pleiotropic effects, conferring phage resistance but increasing sensitivity to antibiotics [49]. This work provides an example of antagonistic pleiotropy occurring within a community, wherein one allele has beneficial fitness effects on one trait and deleterious effects on another. In this case, LPS homolog mutants in *Hafnia* develop resistance to phage TS33 infection while experiencing decreases in total growth in the presence of the fungi. Considering the negative interactions that these fungi are known to have with other microbes, it is possible that the antibiotic/oxidative stress that they apply accounts for the growth defect in LPS mutants growing under these conditions. In other words, genes controlling LPS biosynthesis may have pleiotropic effects in *Hafnia* and lead to trade-offs with evolutionary consequences for *Hafnia* growing in a community of fungi and phage. Moreover, genes involved in the synthesis of enterobacterial common antigen, an extracellular polysaccharide chain found in *Enterobacterales*, have been shown to be important in interactions within increasingly complex microbial communities [50]. It is also possible that antibiotics produced by the fungi may reduce the evolution of phage resistance via LPS modification as this has been shown in *E. coli* [51]. Altogether, these findings suggest a key role of glycan-modified antigens in community interactions.

There is additional evidence that the negative effects of antibiotics on LPS-deficient cells may be genotype-specific. In a study by McGee *et al.*, three of four LPS mutants under study were resistant to both phage and antibiotics of various classes [52]. They observe that *rfaH* and *yciM* (regulating synthesis of Lipid A; [53]) mutants are phage- and antibiotic-resistant; the phage–antibiotic resistance trade-off was observed only in the *rfaP* (facilitating synthesis of the core oligosaccharide) mutant. While we were not able to isolate an *rfaP* mutant for our experiments, our findings do suggest that all LPS mutations may not provide a trade-off within a community context. *manC* is involved in the biosynthesis of GDP-mannose, a key sugar nucleotide precursor used in the synthesis of the O-antigen [54]. The disruption in *manC* was sufficiently deleterious to produce a statistically significant growth deficit in the presence of fungi. In addition, insertion mutants in O-antigen polymerase (*wzy*), O-antigen ligase (*rfaL*), and the antitermination protein that regulates expression of *rfa* genes (*rfaH*) exhibit growth deficits in the presence of the fungi. We expect that mutations in these genes would result in improper polymerization of the sugar residues within the O-antigen, faulty attachment of the O-antigen to the growing LPS, and poor regulation of LPS synthesis. In any case, we would predict that these mutants lacked the ability to assemble and ligate the O-antigen on a whole. Combining our results with those of McGee *et al.*, it is plausible that some Lipid A and the O-antigen mutations are likely implicated in increased susceptibility to antibiotics and other negative interactions with community members. However, the O-antigen is possibly more important than Lipid A for the existence of phage resistant trade-offs when experiencing the aforementioned negative environmental stimuli.

Furthermore, our unique approach to the study of host–phage interactions underscores the critical importance of considering the community in these investigations. Using *Hafnia* as a model, we show that the ecological context can affect the growth outcome of bacteria. The outcome of *Hafnia*-TS33 interactions is more positive when the fungi are absent. Additionally, both *Hafnia* and the phage experience density suppression mediated by the fungi. These results are similar to what is seen in wastewater microbiomes, where protists negatively impact host–phage interactions. Therefore, including all members of the community in our study allowed us to observe the dependence of *Hafnia*–phage interactions on both fungi.

Altogether, our findings shed light on the unique contributions of community members to bacteria–phage interactions and thus reinforce the need to consider the microbiome in studies of the biology of bacteria and phages. Additionally, through this study, we have just begun to understand the role of the LPS in mediating host–phage interactions within a community. Finally, we have introduced a model host–phage interaction within a community context, which we hope will serve as a platform for future investigations of microbial interactions across the three domains of life.

## Supplementary Material

Supplementary_Table_and_Figure_ycae101

## Data Availability

The datasets generated during this work are available on NCBI at the following links: The phage TS33 genome is available at NCBI under Accession Number OR844384. The raw sequencing reads are available under BioProject Number PRJNA1099227. The assembled genome is also available under Accession Number OR844384. *Hafnia* RB-TnSeq library is available under SRA Accession Number SRR26912301 and BioProject PRJNA1043726. Raw *Hafnia* sequencing reads from community growth experiments are available under BioProject PRJNA1043638. Raw Illumina sequencing reads from the genomes of *Hafnia* mutants (Δ*manC*, Δ*wzy*-1, Δ*wzy*-2 and Δ*rfaH*) are available under the following BioProject and SRA Accession Numbers (Whole genome sequences are not available for the Δ*rfaL* mutant): Δ*manC* raw sequencing reads available under BioProject PRJNA1096668 and SRA Accession Number SRR28576124. Δ*wzy*-1 raw sequencing reads available under BioProject PRJNA1096670 and SRA Accession Number SRR28576126. Δ*wzy*-2 raw sequencing reads available under BioProject PRJNA1096673 and SRA Accession Number SRR28576125. Δ*rfaH* raw sequencing reads available under BioProject PRJNA1096963 and SRA Accession Number SRR28576114. The *Hafnia* library characterization file is available on Github at the following link: https://github.com/DuttonLab/hafnia-jb232-library-characterisation.
