# Supplementary material for: Phage resistance mutations affecting the bacterial cell surface increase susceptibility to fungi in a model cheese community
Source: ISME Commun. 2024 Aug 2;4(1):ycae101. doi: 10.1093/ismeco/ycae101 (PMC11409937; doi:10.1093/ismeco/ycae101)
Supplement: Supplementary_Table_and_Figure_ycae101 [file supplementary_table_and_figure_ycae101.zip › Supplementary_Table_and_Figure_ycae101/FigureS1.pdf]

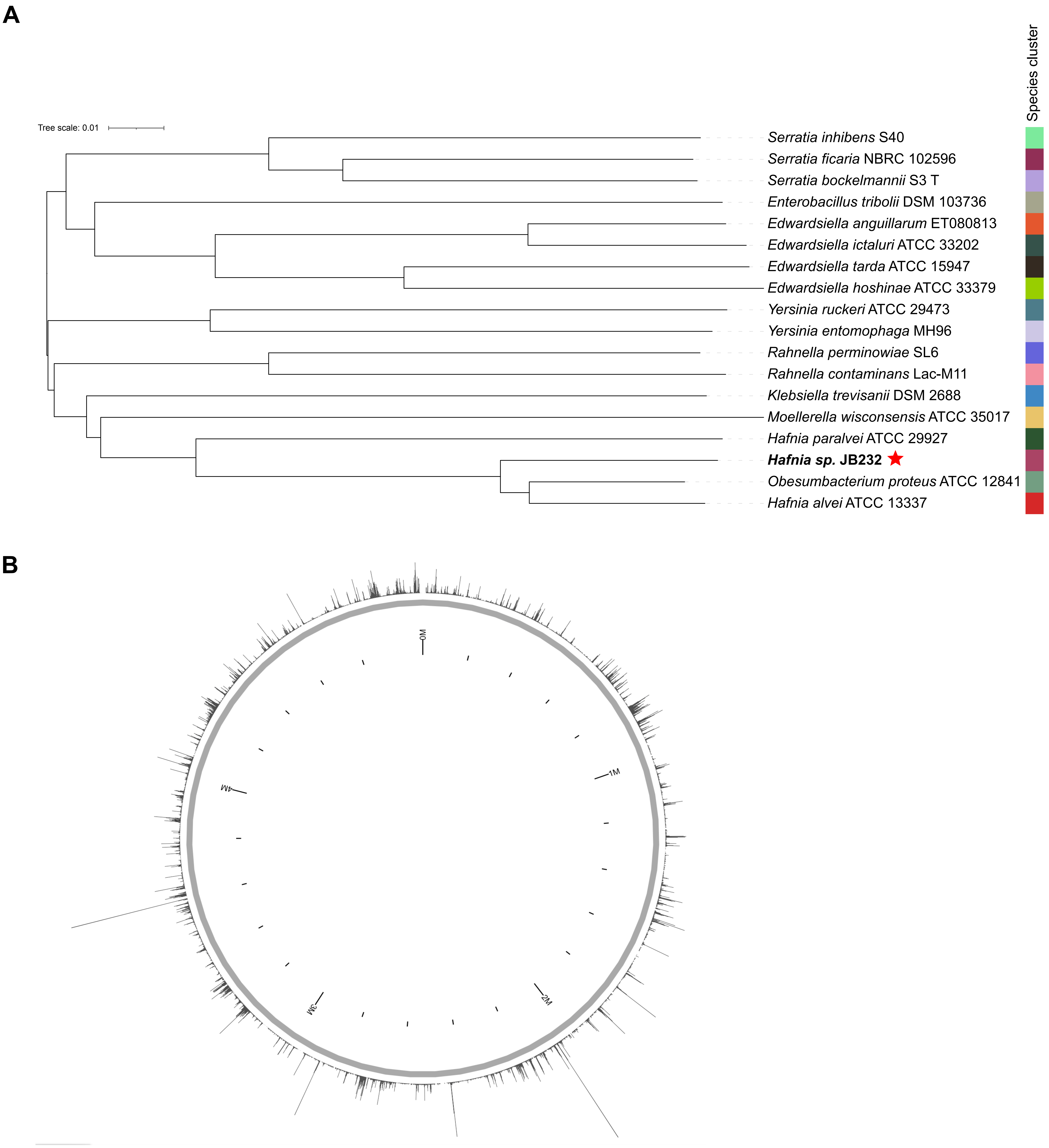
A
Tree scale: 0.01
Species cluster
Serratia inhibens S40
Serratia ficaria NBRC 102596
Serratia bockelmannii S3 T
Enterobacillus tribolii DSM 103736
Edwardsiella anguillarum ET080813
Edwardsiella ictaluri ATCC 33202
Edwardsiella tarda ATCC 15947
Edwardsiella hoshinae ATCC 33379
Yersinia ruckeri ATCC 29473
Yersinia entomophaga MH96
Rahnella perminowiae SL6
Rahnella contaminans Lac-M11
Klebsiella trevisanii DSM 2688
Moellerella wisconsensis ATCC 35017
Hafnia paralvei ATCC 29927
Hafnia sp. JB232
Obesumbacterium proteus ATCC 12841
Hafnia alvei ATCC 13337
B
0M
1M
2M
3M
4M
